# Identification of Novel Proteins Co-Purifying with Cockayne Syndrome Group B (CSB) Reveals Potential Roles for CSB in RNA Metabolism and Chromatin Dynamics

**DOI:** 10.1371/journal.pone.0128558

**Published:** 2015-06-01

**Authors:** Serena Nicolai, Silvia Filippi, Manuela Caputo, Lubos Cipak, Juraj Gregan, Gustav Ammerer, Mattia Frontini, Daniela Willems, Giorgio Prantera, Adayabalam S. Balajee, Luca Proietti-De-Santis

**Affiliations:** 1 Unit of Molecular Genetics of Aging, Department of Ecology and Biology, University of Tuscia, 01100, Viterbo, Italy; 2 Cancer Research Institute, Slovak Academy of Sciences, Bratislava, Slovak Republic; 3 Department of Genetics, Comenius University in Bratislava, Slovakia; 4 Department of Biochemistry, Mass Spectrometry Facility, Max F. Perutz Laboratories, University of Vienna, Vienna, Austria; 5 Department of Haematology, University of Cambridge, CB2 0PT, Cambridge, United Kingdom; 6 Center for Radiological Research, Department of Radiation Oncology, Columbia University Medical Center, New York, New York, 10032, United States of America; Universita' di Milano, ITALY

## Abstract

The CSB protein, a member of the SWI/SNF ATP dependent chromatin remodeling family of proteins, plays a role in a sub-pathway of nucleotide excision repair (NER) known as transcription coupled repair (TCR). *CSB* is frequently mutated in Cockayne syndrome group B, a segmental progeroid human autosomal recessive disease characterized by growth failure and degeneration of multiple organs. Though initially classified as a DNA repair protein, recent studies have demonstrated that the loss of CSB results in pleiotropic effects. Identification of novel proteins belonging to the CSB interactome may be useful not only for predicting the molecular basis for diverse pathological symptoms of CS-B patients but also for unraveling the functions of CSB in addition to its authentic role in DNA repair. In this study, we performed tandem affinity purification (TAP) technology coupled with mass spectrometry and co-immunoprecipitation studies to identify and characterize the proteins that potentially interact with CSB-TAP. Our approach revealed 33 proteins that were not previously known to interact with CSB. These newly identified proteins indicate potential roles for CSB in RNA metabolism involving repression and activation of transcription process and in the maintenance of chromatin dynamics and integrity.

## Introduction

Cockayne syndrome (CS) is an autosomal recessive disorder with shunted growth and developmental defects involving a wide range of tissues and organs [1]. Among these, progressive neurological abnormalities including demyelination, ataxia, and cerebellar atrophy are the hallmarks of this syndrome. Two genes (*CSA and CSB*) have been implicated in the pathogenesis of Cockayne syndrome and patients with mutations in *CSB* are more frequent [2, 3]. The CSB is a member of the SWI/SNF ATP dependent chromatin remodeling family of proteins [4] and plays a role in transcription-coupled repair (TCR), an important sub-pathway of nucleotide excision repair (NER) [5–7]. Patients afflicted with CS exhibit multiple pathological symptoms and some of which may not be solely explained by DNA repair defects. This has led to the hypothesis that CSB has additional roles in processes other than the TCR pathway [7–10]. Consistent with this notion, CSB protein has been shown to interact and stimulate the transcriptional complexes of all the three nuclear RNA polymerases (I, II and III), indicating a regulatory role for CSB in basal transcription [11–18]. Additionally, CSB has been demonstrated to be a key regulator of p53 pathway [19–22]. Unraveling additional novel functions of CSB is likely to provide useful mechanistic insights for the diverse pathological symptoms of CS-B patients.

It is becoming increasingly obvious that protein-protein interactions are vital for the efficiency of numerous biological processes including DNA replication, transcription, repair and recombination. Identification of interacting proteins or protein complexes has often proved valuable in deducing the novel functions of any given gene of interest. Although CSB has been shown to interact with some of the functional units of basal transcription factor TFIIH and RNA polymerase elongation complexes, identification of additional proteins and protein complexes interacting with CSB may help in unraveling some of the yet unidentified functions of CSB which may be relevant for defining the phenotype-genotype correlation of CSB patients. With this objective to identify proteins co-purifying with CSB, tandem affinity purification (TAP) tag technology coupled with mass spectrometry was employed in this study. TAP technique is often used to isolate proteins that can be identified by subsequent mass-spectrometry analysis [23–25]. In our approach, a TAP tag was fused with *CSB* cDNA and the construct was subsequently transfected into suitable host cells (CSIAN cells) and the functionality of the CSB-TAP protein was verified by complementation of UV sensitivity. Proteins associated with CSB-TAP fusion protein were then isolated through two sequential affinity purification steps. Finally, the isolated proteins were size fractionated on SDS polyacrylamide gels and analyzed by mass spectrometry essentially as described before [26, 27].

## Results

### Establishment of stable cell lines expressing CSB-TAP protein

**Fig 1:**
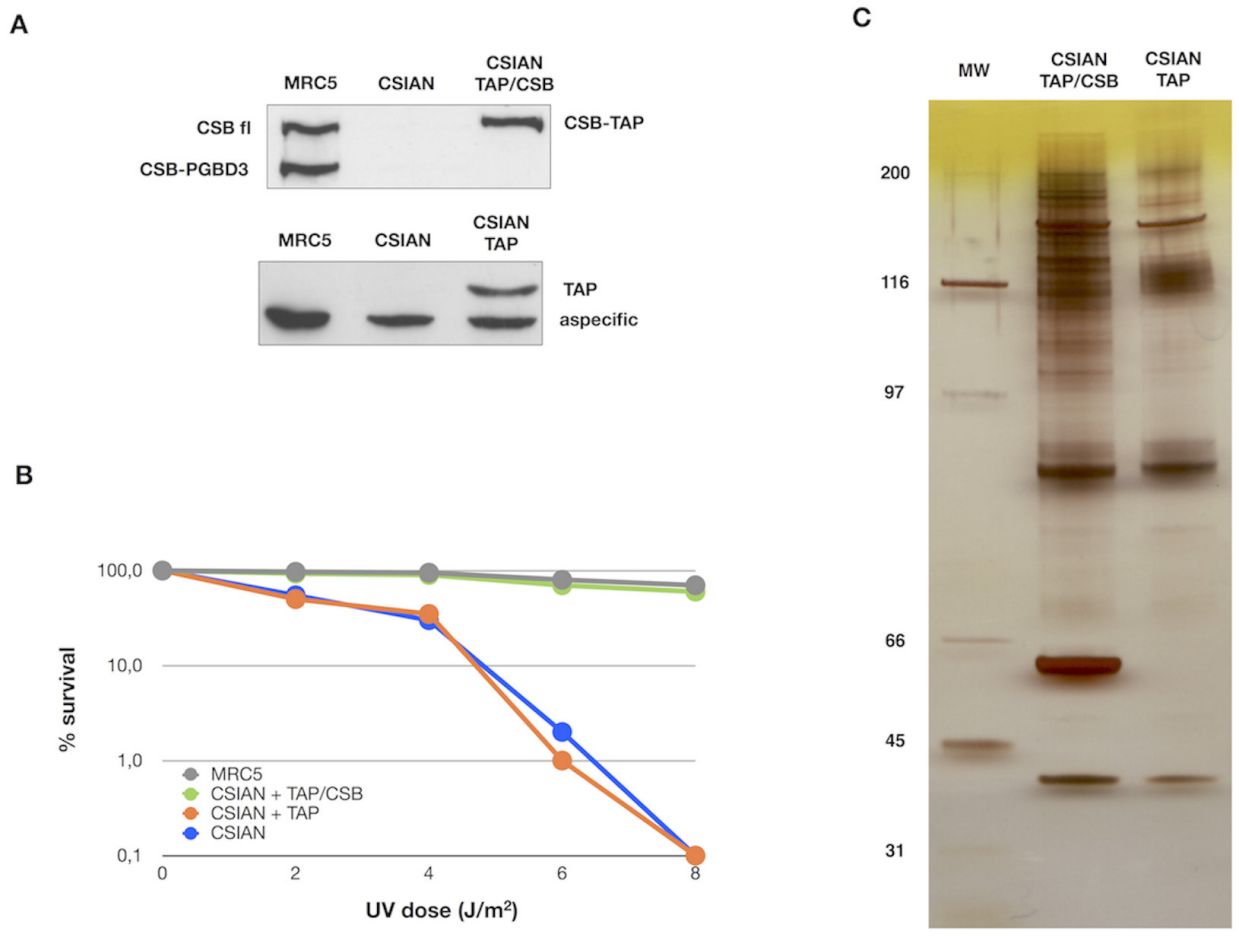
Establishment of stable cell lines expressing CSB-TAP protein and Identification of proteins that co-purified with CSB-TAP. (A) Western blot showing the expression of endogenous CSB full-length (CSB fl) and CSB-PGBD3 proteins in wild type MRC5 fibroblasts and either chimeric CSB-TAP protein (CSIAN TAP/CSB) or TAP domain (CSIAN TAP) in CSB-deficient fibroblasts CSIAN after stable transfection. TAP tagged proteins were detected using rabbit polyclonal anti TAP tag (CAB 1001, Pierce) and endogenous CSB, either fl and PGBD3 isoforms, using rabbit polyclonal anti-ERCC6 (H300, Santacruz). (B) UV survival demonstrated the complementation of UV survival in CSB-TAP transfected cells. Clonogenic survival after UV exposure in MRC5, CSIAN and isogenic clonal populations of CSIAN transfectant cell lines is shown as the percentage of survival. Results shown are the average values of three independent experiments. (C) Silver staining of proteins associated with CSB-TAP and TAP that were isolated by tandem affinity purification and, separated on a 4–12% Bis-Tris gel.

CSIAN (CSB deficient) cells were transfected with either pZome-1-N (mock), or pZome-1-N-TAP-CSB for stable expression of CSB-TAP tagged protein. A well-established UV sensitive CSB deficient cell line (CSIAN) was chosen for our study because it would allow us to verify the functionality of the CSB-TAP protein by genetic complementation. CSIAN cells stably expressing TAP-CSB or TAP alone were selected with puromycin. The selected clones were amplified and screened for the expression of TAP-tagged CSB protein. To avoid the possible artifacts arising from CSB-TAP over expression, a stably transfected clonal cell line was chosen whose CSB expression level was similar to the endogenous CSB level observed in CSB proficient MRC5 cells (Fig 1A). Determination of cell survival after UV exposure demonstrated that the CSB-TAP fusion protein was functional and the cell survival was substantially enhanced in CSB-TAP transfected CSIAN cells relative to vector alone-transfected CSIAN cells. Further, cellular resistance to UV in CSB-TAP fusion protein expressing CSIAN cells was almost identical to CSB proficient wild type MRC5 cells (Fig 1B).

After ascertaining the functionality of transfected CSB-TAP fusion protein, CSB-TAP and TAP transfected cells were grown and the total cellular proteins were extracted from approximately 1X10^10^ cells were utilized for TAP affinity purification. Fig 1C shows a representative silver stained gel of CSB-TAP co-purifying proteins (CSIAN TAP/CSB) that were subsequently analyzed by mass spectrophotometry. Proteins isolated from TAP vector alone (CSIAN TAP) served as negative control. As showed in panel C, a distinct pattern of purified proteins was observed in CSB-TAP expressing cells in comparison to TAP tag expressing cells. To facilitate the identification of purified proteins, each lane of the SDS-acrylamide gel containing the size-fractionated proteins was cut into 40 slices and the slices were subjected to mass-spectrometry analysis.

### Identification of proteins that co-purified with CSB-TAP

**Fig 2:**
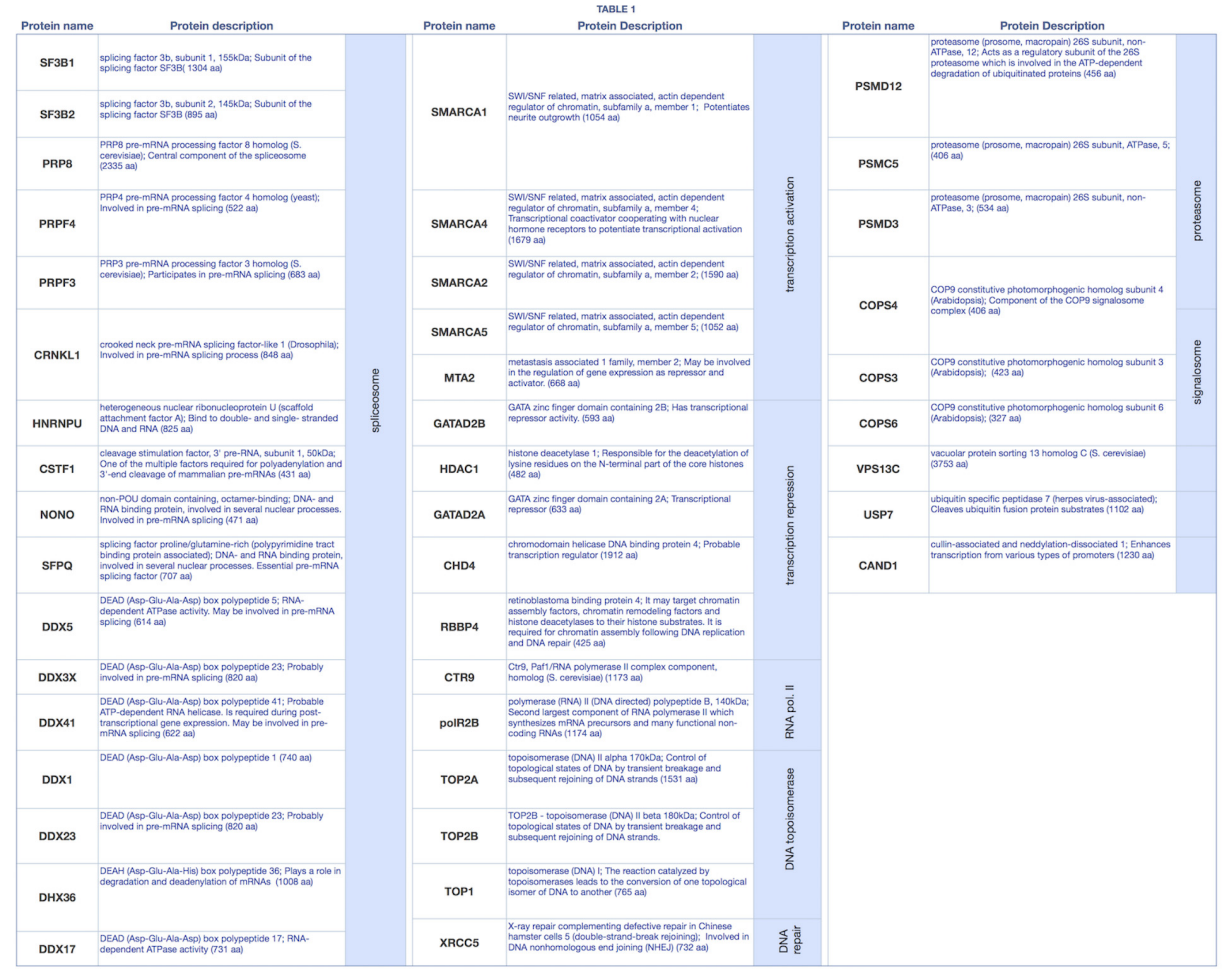
Names of proteins co-purifying with CSB-TAP fusion protein and their associated biological processes.

**Fig 3:**
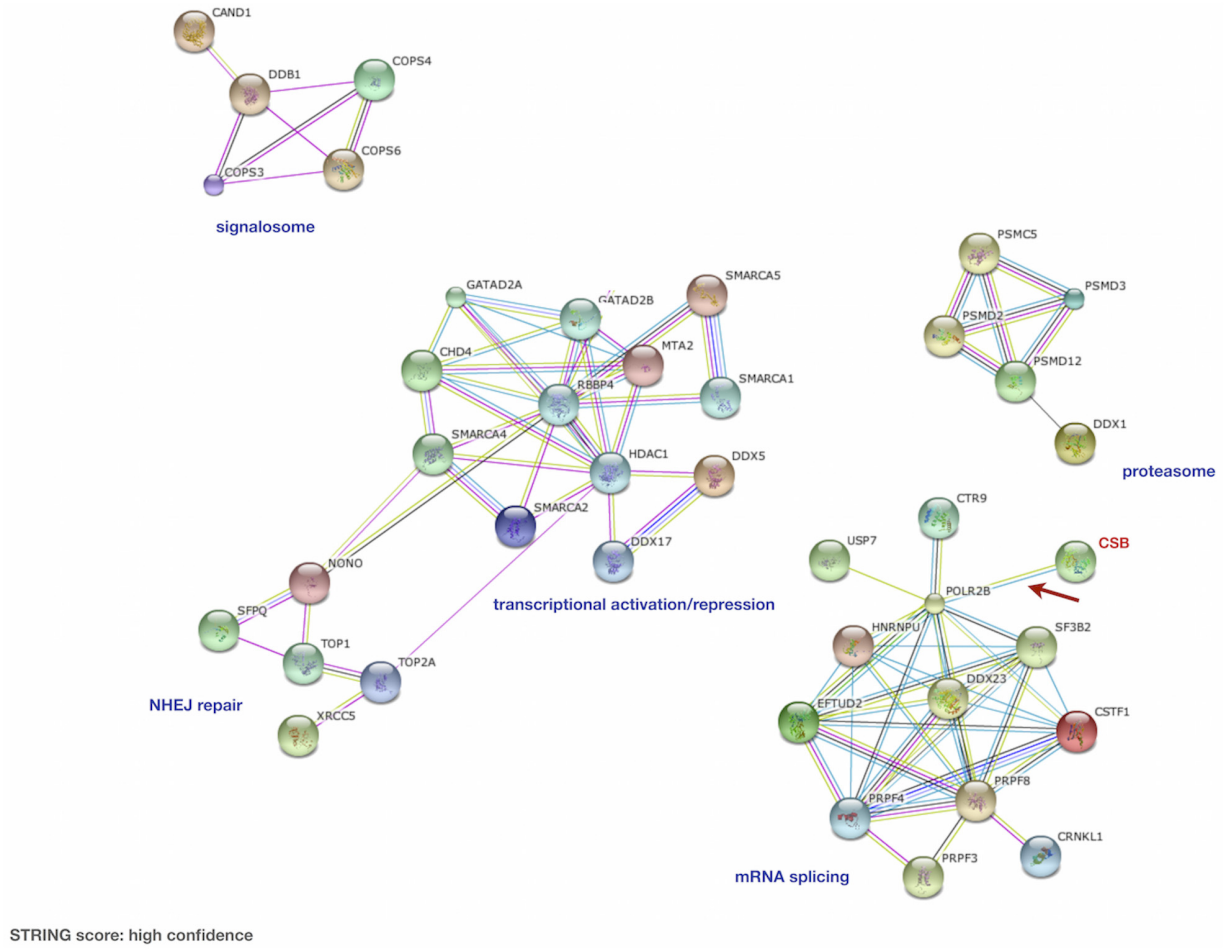
Interactome (protein-protein interaction) of CSB-associated proteins in CSIAN cells. The protein-protein interaction network was constructed using String software online (version 8.3, www.string-db.org) for CSB-TAP co-purifying proteins. Blue dotted line area represents the RNA splicing cluster. The red dotted line area represents the NHEJ repair cluster; the green dotted line area represents the gene expression regulators cluster and the grey dotted line area the proteasome cluster.

Tandem affinity purification and mass spectrometry analyses identified proteins that co-purified with CSB-TAP fusion protein, but were absent in the purification from control cells expressing TAP tag alone (S1 Table). Fig 2 summarizes the names and the biological processes of proteins co-purifying with CSB-TAP fusion protein. The String software (Fig 3) was used to reveal CSB-associated proteins based on their known and predicted protein interactions. Apart from the established interaction of CSB with RNA pol II, CSB association was found with five major protein clusters. More then 40% of these proteins (19 out of 45) have demonstrated roles either in general aspects of RNA processing or more specifically in RNA splicing. Some of these proteins are essential human splicing factors such as snRNP subunits and some are proteins anchoring snRNPs during pre-mRNA splicing for aligning and cleavage stimulation. Others have functions closely associated with RNA splicing. Multiple members of the DEAD-box helicase family, which are thought to control RNA base-pairing interactions at different stages of spliceosome assembly and catalysis, have been identified as CSB-TAP co-purifying proteins. In addition to splicing and RNA-processing factors, a group of proteins involved in the repression/relieving of transcription process were also identified: SMARCA 1, SMARCA2, SMARCA4 and SMARCA5; belonging to the SWI/SNF related, matrix associated, actin dependent regulator of chromatin and helicase-like transcription factor (HLTF), all of which are known to participate in transcriptional activation by favoring ATP dependent chromatin remodeling processes. Other CSB-TAP co-purifying proteins include metastasis associated family 1, member 2 (MTA2) which can function both as a transcriptional repressor and activator, and 3 proteins acting as transcriptional repressors such as HDAC1 (histone deacetylase 1), and GATA2A and GATA2B (GATA zinc finger domain containing 2A or 2B). In addition to transcription factors, our study also revealed Topoisomerases I and II, as well as XRCC5 as CSB-TAP co-purifying proteins. These factors have crucial roles in DNA mediated metabolic activities such as replication, repair and recombination. In addition, a number of proteins belonging to the proteasome (PSMD2, PSMD3, PSMD12 and PSMC5) and to the signalosome (COPS3, COPS4, COPS6) also co-purified with CBS-TAP. Taken together, the identification of proteins co-purifying with CSB-TAP provide novel insights for diversified roles of CSB in global transcription process and chromatin dynamics.

### Validation of CSB interacting proteins by reciprocal co-immunoprecipitation

Co-immunoprecipitation (co-IP) is the most widely used *in vitro* method for verification of protein-protein associations identified with other techniques [28–30]. To verify and validate the data obtained by TAP-tag experiments, immunoprecipittation was performed utilizing the total cellular proteins prepared from CSIAN cells cotransfected with plasmids expressing either Flag-tagged CSB or Myc-tagged gene for each of the 36 out of 45 CSB interacting proteins identified by CSB-TAP-tag assay. Immunoprecipitation was performed either with anti-Flag or anti-Myc antibody. Flag and Myc-immunoprecipitates were analyzed by western blot using either Myc- or Flag-antibodies.

**Fig 4:**
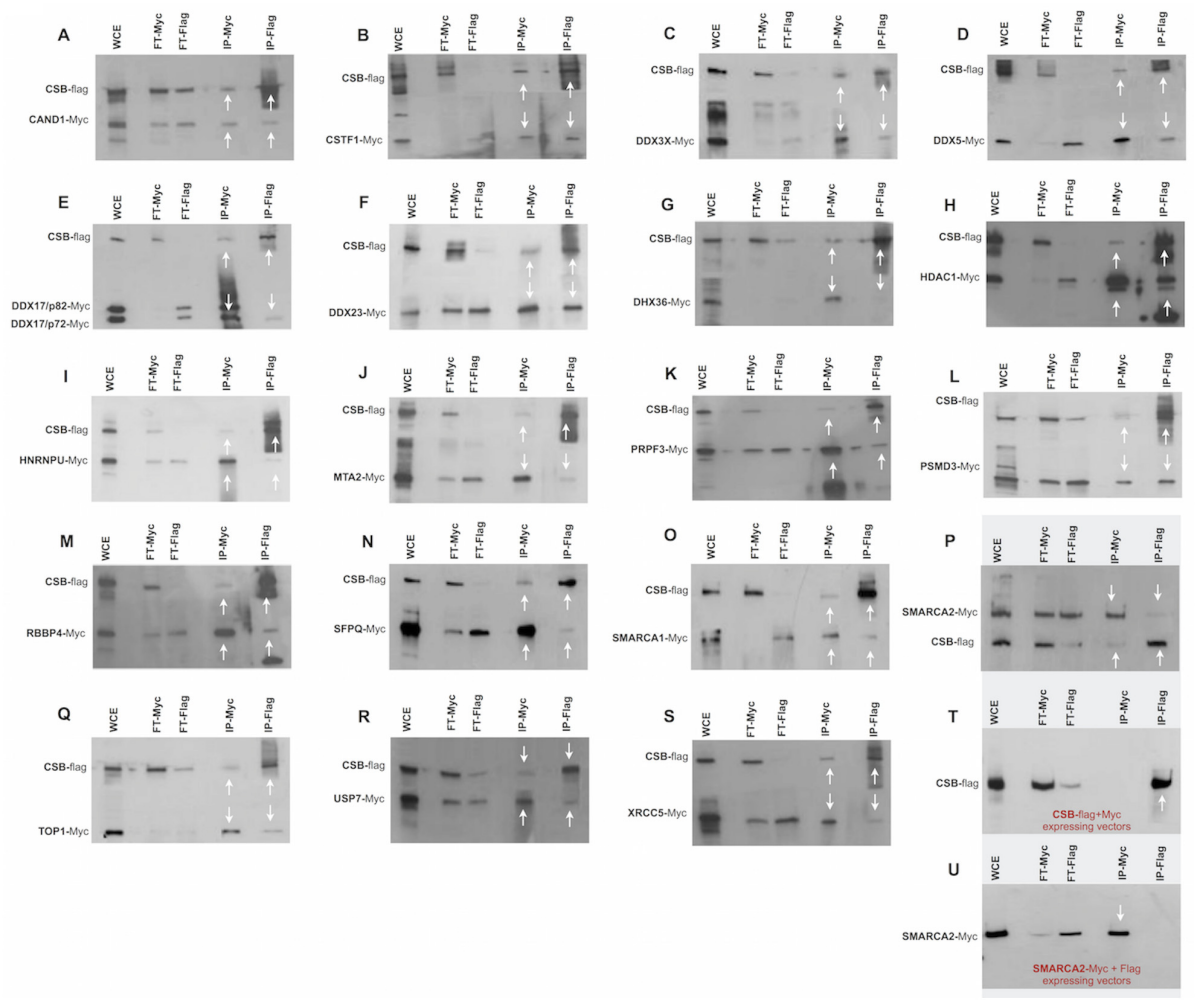
Co-immunoprecipitations studies. Co-immunoprecipitation was performed using the lysates of CSIAN cells, transiently expressing flag tagged CSB and myc-tagged proteins of interest. Whole cell extracts, prepared 24 hr post transfection, were immunoprecipitated for either CSB or putative interacting proteins using respectively Flag or c-Myc monoclonal antibod**y** conjugated agarose resins, followed by Western blot analysis using anti-myc and anti-flag antibodies respectively. * two isoforms p72 and p82 arise from ddx17 gene through the use of different in-frame translation initiation codons. (WCE, whole cellular extract; FT, Flow-Through; IP, immunoprecipitation).

**Fig 5:**
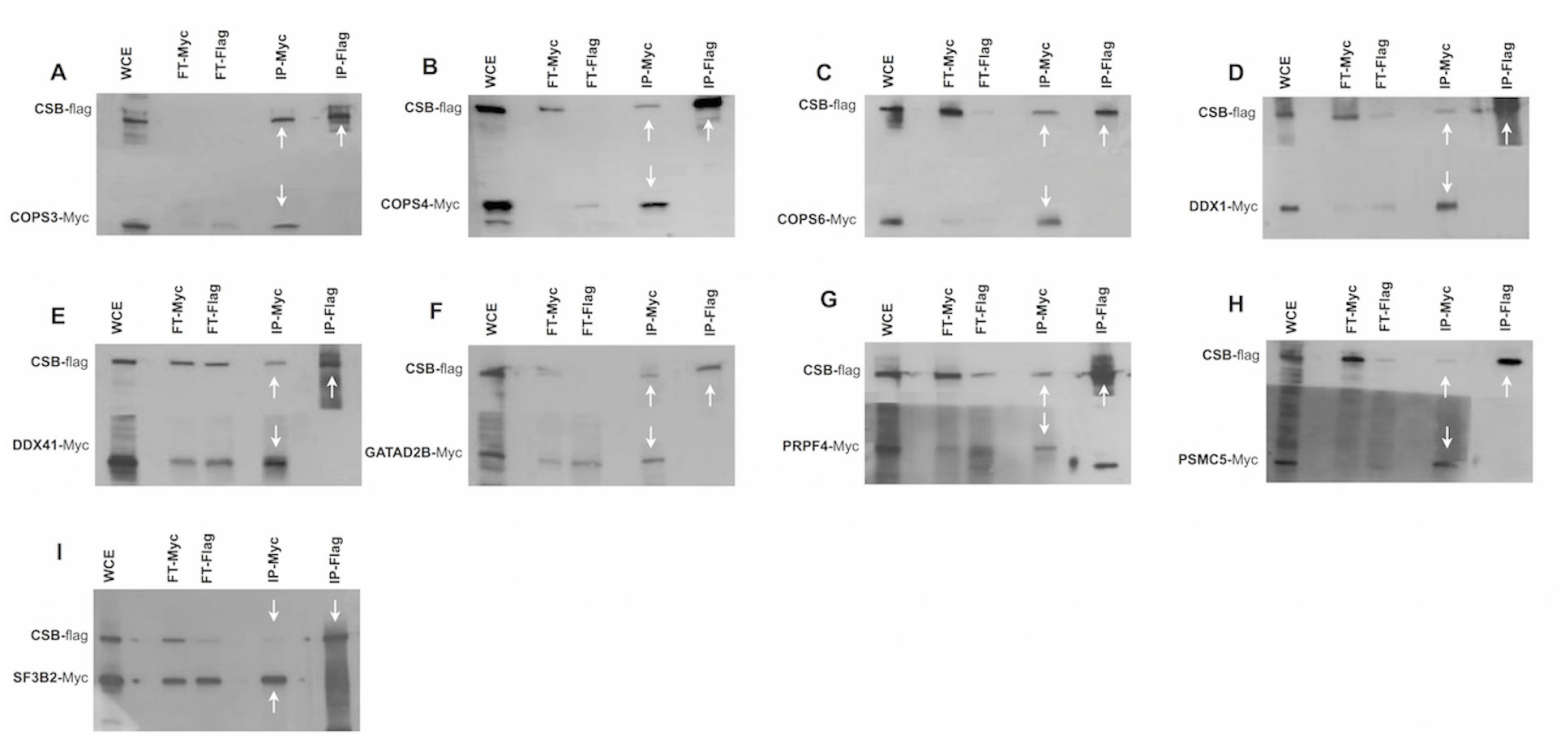
Co-immunoprecipitations studies. Co-immunoprecipitation was performed using the lysates of CSIAN cells, transiently expressing flag tagged CSB and myc-tagged proteins of interest. Whole cell extracts, prepared 24 hr post transfection, were immunoprecipitated for either CSB or putative interacting proteins using respectively Flag or c-Myc monoclonal antibod**y** conjugated agarose resins, followed by Western blot analysis using anti-myc and anti-flag antibodies respectively. (WCE, whole cellular extract; FT, Flow-Through; IP, immunoprecipitation).

**Fig 6:**
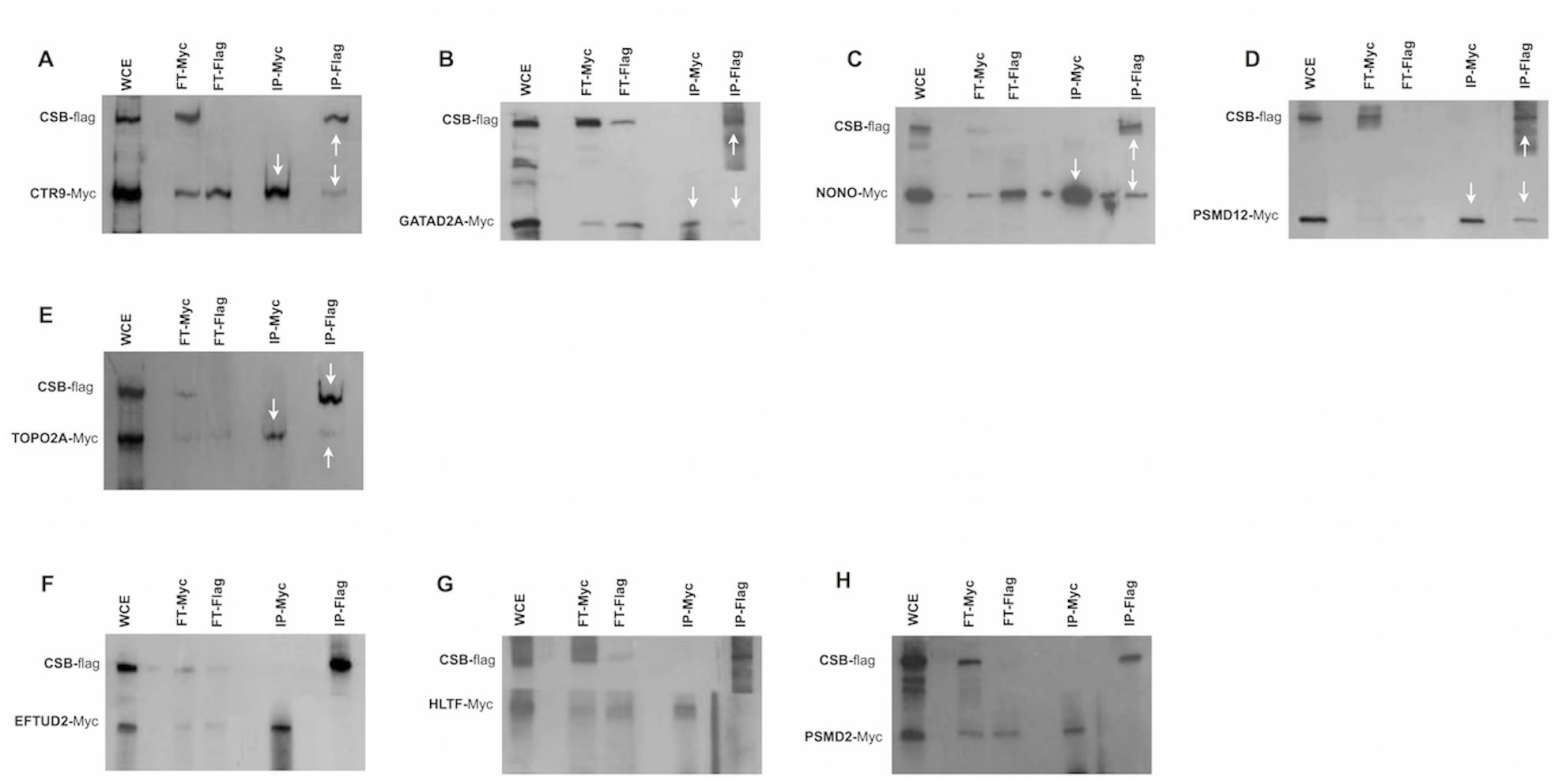
Co-immunoprecipitations studies. Co-immunoprecipitation was performed using the lysates of CSIAN cells, transiently expressing flag tagged CSB and myc-tagged proteins of interest. Whole cell extracts, prepared 24 hr post transfection, were immunoprecipitated for either CSB or putative interacting proteins using respectively Flag or c-Myc monoclonal antibod**y** conjugated agarose resins, followed by Western blot analysis using anti-myc and anti-flag antibodies respectively. (WCE, whole cellular extract; FT, Flow-Through; IP, immunoprecipitation).

Immunoprecipitation studies confirmed the reciprocal interaction of CAND1, CSTF1, DDX3X, DDX5, DDX17, DDX23, DHX36, HDAC1, HNRNPU, MTA2, PRPF3, PSMD3, RBBP4, SFPQ, SMARCA1, SMARCA 2, TOP1, USP7 and XRCC5 proteins with CSB when the IP was performed using antibodies either for Flag or for Myc. (Fig 4A–4S). Moreover we established that COPS3, COPS4, COPS6, DDX1, DDX41, GATAD2B, PRPF4, PSMC5 and SF3B2 co-precipitated with CSB only when the IP was performed using Myc antibody but not with Flag antibody (Fig 5A–5I). Likewise, CTR9, GATA2A, NONO, PSMD12, and TOPO 2A co-precipitated with CSB when the IP was performed using Flag antibody but not when using the Myc antibody (Fig 6A–6E). Interaction of the above-mentioned proteins with CSB detected only with Flag antibody but not with Myc and *vice versa* is likely due to several reasons with the most probabale one is the differential masking of antibody binding site owing to interactions with proteins or protein complexes. In some co-IPs, the intensity of the band representing the interacting protein is of very low intensity. In some cases, such as SMARCA2 (Fig 4P), for instance, the intensity of the band representing the interacting protein is low in both reciprocal IPs. In order to verify that the detection of weakly interacting protein(s) with CSB is not due to non-specific background binding to the agarose beads under our experimental conditions, co-IP experiments were repeated including negative controls. As visualized in Fig 4T–4U, IP against Myc failed to precipitate CSB protein when Myc is not fused to SMARCA2; as well IP against Flag failed to precipitate SMARCA2 when the Flag epitope was not fused to CSB. These two control experiments clearly demonstrates that the bands, though of low intensity, reflect a true interaction with CSB and are not due to non-specific background binding to the agarose beads.

A total of three proteins (EFTUD2, HLTF and PSMD2) identified by CSB-TAP assay failed to show interaction with CSB by IP with both of the antibodies (Fig 6F–6H) illustrating that their interaction with CSB may be either weak or indirect through other proteins. In summary, a total of 33 novel protein–protein interactions identified in the TAP-tag screen were confirmed by co-IP. Since we assessed the interactions in the absence of exogenous DN damage, additional studies are certainly warranted to decipher the functional relevance of these interactions in the context of DNA repair activities.

## Discussion

CS patients exhibit a multitude of symptoms spanning from shunted growth to complex neurological abnormalities. Although a crucial role for CSB has been demonstrated in exogenously induced TCR pathway, it has remained enigmatic as to whether a deficiency in TCR alone can fully account for some of the pathological symptoms such as shunted growth and multiple organ/tissue degeneration in CSB patients. Elucidation of novel additional functions of CSB can provide molecular insights into the complex pathological phenotypes of CSB patients. Since protein-protein interaction is crucial for many of the biological processes, identification of novel interacting partners for CSB may aid in deducing the potential novel functions of CSB.

In this study, the TAP-tag technology coupled with mass spectrometry was used to identify novel proteins co-purifying with CSB-TAP. Using this approach, a total of 33 novel CSB interacting proteins were identified and their interaction with CSB was further verified and validated by co-immunoprecipitation studies using Flag and Myc antibodies. The identified proteins were grouped based on their interactions by the String software and we found two most interesting and significant clusters that contain proteins involved in mRNA splicing and chromatin remodeling. In our opinion, this study is the first and fore most revealing the potential link between CSB and some of the RNA splicing factors. The fact that CSB-TAP co-purified with both RNA polymerase elongation complex and splicing complex indicates that CSB can potentially regulate the overall efficiency of transcription machinery through its associated protein complexes. It is well known that RNA elongation and mRNA splicing steps are both molecularly interlinked and both of these intrinsic processes are needed for transcription efficiency [31]. It is possible that CSB regulates both of these steps in transcription process through its interaction with some of the key proteins. Assuming that CSB is critical for transcription machinery, its loss is expected to be detrimental for tissue viability and homeostasis. In support of this notion, a recent study has suggested that CS is caused by transcriptional deregulation of genes involved in growth suppressive, inflammatory and pro-apoptotic pathways [32]. One of the novel aspects of our study is the demonstration that CSB co-purified with some of the important RNA splicing factors.

It is well known that some of the post-transcriptional modifications involving 5’ end capping, 3’- polyadenylation and mRNA splicing are crucial not only for the precise transcriptional regulation of any given gene but also for the functional stability of the transcriptome [33, 34]. Therefore, defects in any of these fundamental steps can disrupt the translation process, thereby affecting many biological processes. Additionally, aberrant splicing has been described as a predisposing factor for a number of human diseases [35]. One such disease is retinitis pigmentosa (RP), which results in progressive retinal degeneration and RP is also a prominent clinical feature of CS. Interestingly, we demonstrated here that two of the three genes responsible for autosomal dominant RP, PRPF3 and PRPF4 [36] co-purified with CSB-TAP. An other splicing factor, HNRNPU, which we identified to co-purify with CSB-TAP, when mutated gives rise to clinical features that are shared by CS patients: microcephaly, developmental delay, hearing loss, vertebral anomalies and characteristic facial features [37, 38]. Future studies are certainly required to verify and validate whether RNA splicing is affected by CSB deficiency and whether RNA splicing defects lead to pleiotropic effects observed in CS patients.

Chromatin remodeling is a prerequisite for all types of DNA metabolic activities involving replication, transcription, repair and recombination and it is a dynamic ATP dependent process through which the highly condensed chromatin is made accessible to the factors for DNA metabolic activities. Additionally, chromatin-remodeling events are critical for preserving the epigenome integrity of both multipotent adult stem and differentiated cells in a tissue microenvironment. The importance of these processes is also being well recognized in human aging [39]. Since the initial reports of the involvement of CSB in RNA pol II elongation [10, 11], numerous studies have documented the critical importance of CSB both in initiation and elongation of transcription by RNA polymerases I, II and III in the presence or absence of endogenous and exogenous DNA damage. Further, interaction of CSB with some of the subunits of the basal transcription factor TFIIH and RNA polymerase II has already been demonstrated [40]. Consistent with it, we observed that two subunits of RNA polymerase II (RPB2 and CTR9) co-purified with CSB Further, CSB functions as an elongation factor for small structured RNA and loss of CSB causes the metaphase fragility of human U1, U2 and 5S genes [41].

CSB belongs to yeast SWI/SNF family of proteins, which have demonstrated roles in chromatin remodeling [4, 42]. In this study, we have identified 4 most prominent chromatin-remodeling factors (SMARCA1, SMARCA2, SMARCA4 and SMARCA5) and all of these belong to SWI/SNF family of proteins [43]. These proteins belong to neural progenitors specific chromatin remodeling complex (npBAF complex) and neuron-specific chromatin remodeling complex (nBAF complex) [44]. The npBAF complex is essential for self-renewal/proliferative capacity of multipotent neural stem cells. The nBAF complex together with CREST plays a role regulating the activity of genes essential for dendrite growth. Co-purification of CSB-TAP with components of npBAF and nBAF complex indicates that CSB may play a crucial role in preserving the functional integrity of different brain cell types (neural stem/progenitor cells, neurons and glial cells) through successful co-ordination of transcription and chromatin remodeling activities. Therefore, loss of these functions due to CSB deficiency is likely to disrupt the tissue homeostasis in brain leading to neurological abnormalities. However, these interesting possibilities largely remain to be experimentally validated. Some of these proteins (SMARCA) are also required for the co-activation of estrogen responsive promoters and vitamin D-coupled transcription regulation via its association with the WINAC complex. As already described, deficiencies in hormonal response may account for shunted growth and skeletal abnormalities frequently observed in CSB patients [17].

Strikingly, all of the chromatin remodeling factors (SMARCA1, SMARCA2, SMARCA4 and SMARCA5) are shown to be differentially expressed in diverse cancer cell types. SMARCA1, also known as nucleosome-remodeling factor NURF 140 interacts with CSB but the functional significance of this interaction in the context of CS symptoms remains to be explored. It is also not clear whether CSB regulates the expression levels of SMARCA1, SMARCA2, SMARCA4 and SMARCA5 genes. If CSB loss leads to deregulated expression of chromatin remodeling factors, then most of the developmental defects of CS patients can be explained on the basis of transcription deficiency superimposed on impaired chromatin remodeling dynamics. Interestingly, these deficiencies can also contribute to TCR defect observed in CSB cells because repair process requires efficient chromatin remodeling events to facilitate the rapid accessibility and assembly of repair factors at the sites of DNA damage. Further, UV induced NER occurs at the same rate throughout the genome of CSB deficient cells without any structural bias towards transcriptionally active genes in general and transcribing strand in particular. It is possible that CSB mediates efficient chromatin remodeling events required for TCR through recruitment and assembly of some of the chromatin remodeling factors that are identified in this study. Alternatively, CSB mediated chromatin remodeling events may play a critical role in TCR by efficiently switching “transcription poised” TFIIH to “repair poised” TFIIH [45]. Identification of histone deacetylase 1 (HDAC1) as a protein co-purifying with CSB raises certain interesting hypotheses because HDAC1 is crucial for deacetylation of lysine residues at the N-terminus of core histone proteins H2A, H2B, H3 and H4 [46]. Further, histone deacetylation is instrumental for transcriptional regulation, cell cycle progression and developmental aspects. However, we do not know whether defects in histone deacetylation lead to developmental defects in CS patients. Proteins belonging to GATA family of proteins with zinc finger binding motifs have been recognized as regulators of transcription particularly in hematopoietic cells of both murine and human origin [47]. Therefore, any perturbations in the expression of these genes may lead to compromised immunoresponse. It has been demonstrated that overexpression of GATA2 overexpression promotes proliferation at the cost of differentiation leading to an imbalance between quiescent and replicative cells in a given tissue/organ. It is not currently clear whether or not CSB directly regulates the expression of these genes. Taken together, our identification of CSB-TAP co-purifying proteins in RNA splicing and chromatin remodeling processes suggests that CSB has multifaceted roles in transcription and chromatin dynamics. In our opinion, some of the developmental defects observed in CSB patients can be attributed to deficiencies in chromatin dynamics superimposed on basal transcription.

In addition to mRNA splicing and chromatin remodeling factors, our study identified Topoisomerase I and II (TOP1 and 2 respectively) as well as XRCC5 as CSB-TAP co-purifying proteins. XRCC5 in association with TOP1 plays a role in non homologous end joining (NHEJ) repair pathway for DNA double strand breaks (DSB) which are either spontaneously generated during replication fork stalling or by exposure to agents such as ionizing radiation (IR) [48]. It is interesting to note that CSB deficient cells are moderately sensitive to DSB generated by IR and topoisomerase inhibitors but the contributing factor is not clearly elucidated [49]. Although, we have not assessed whether or not the interaction of above mentioned repair proteins with CSB is increased upon DNA damage, our finding suggests the possibility that CSB may enhance DSB repair fidelity through its interaction with TOP1, 2 and XRCC5. Alternatively, CSB through its interaction with chromatin remodeling factors can also enhance DSB repair efficiency by increasing the chromatin accessibility to DSB repair factors. Besides the above mentioned repair factors, SFPQ and NONO were also found to co-purify with CSB. SFPQ, a component of the spliceosome and U5.4/6 snRNA complexes, interacts with TOP1 and modulates its function [50]. It stimulates dissociation of TOP1 from DNA after cleavage and enhances its translocation between separate DNA helices *in vitro*. Further, SFPQ-NONO heterodimer in combination with TOP1 and XRCC5 participates in NHEJ pathway. This complex in association with Ku70/G22P1-Ku80/XRCC5 (Ku) dimer strongly stimulates DNA end joining repair through direct binding to DNA substrates [51, 52]. Strikingly, DDX1 (DEAD Box 1) was also found to co-purify with CSB-TAP. DDX1, which forms foci at ionizing radiation induced DSB, possesses RNase activity as well as unwinding activity for RNA-DNA and RNA-RNA duplex [53]. DDX1 through its RNase activity removes RNA at DSB sites thereby facilitating the repair in actively transcribing genomic regions. Foci formation of DDX1 is shown to be dependent on the functional status of ATM kinase but it remains to be seen whether or not CSB is essential for the DSB repair function of DDX1.

Finally two clusters related to the signalosome and proteasome related proteins were also found in our experiments, which are consistent with the previously established functions of CSB. It has been recently demonstrated that CSB is in a complex with CSA/DDB1, p300 and COP9 signalosome on lesion-stalled RNA polymerase II [54]. In this complex, CSB is supposed to enhance the efficiency for the incision and excision steps of NER [55]. This function presumably requires the ubiquitin-binding domain (UBD) of CSB. Our recent data showed that CSB is part of an ubiquitin complex which also contains CSA and Mdm2 that modulate ubiquitination/degradation of p53. Interestingly, proteasome defect has been reported in neurodegenerative disorders including premature aging diseases [56]. Also, CUL7, which was found to interact with CSB in this study, plays a role in ubiquitination and mutation in CUL7 gives rise to intrauterine growth retardation [57].

**Fig 7:**
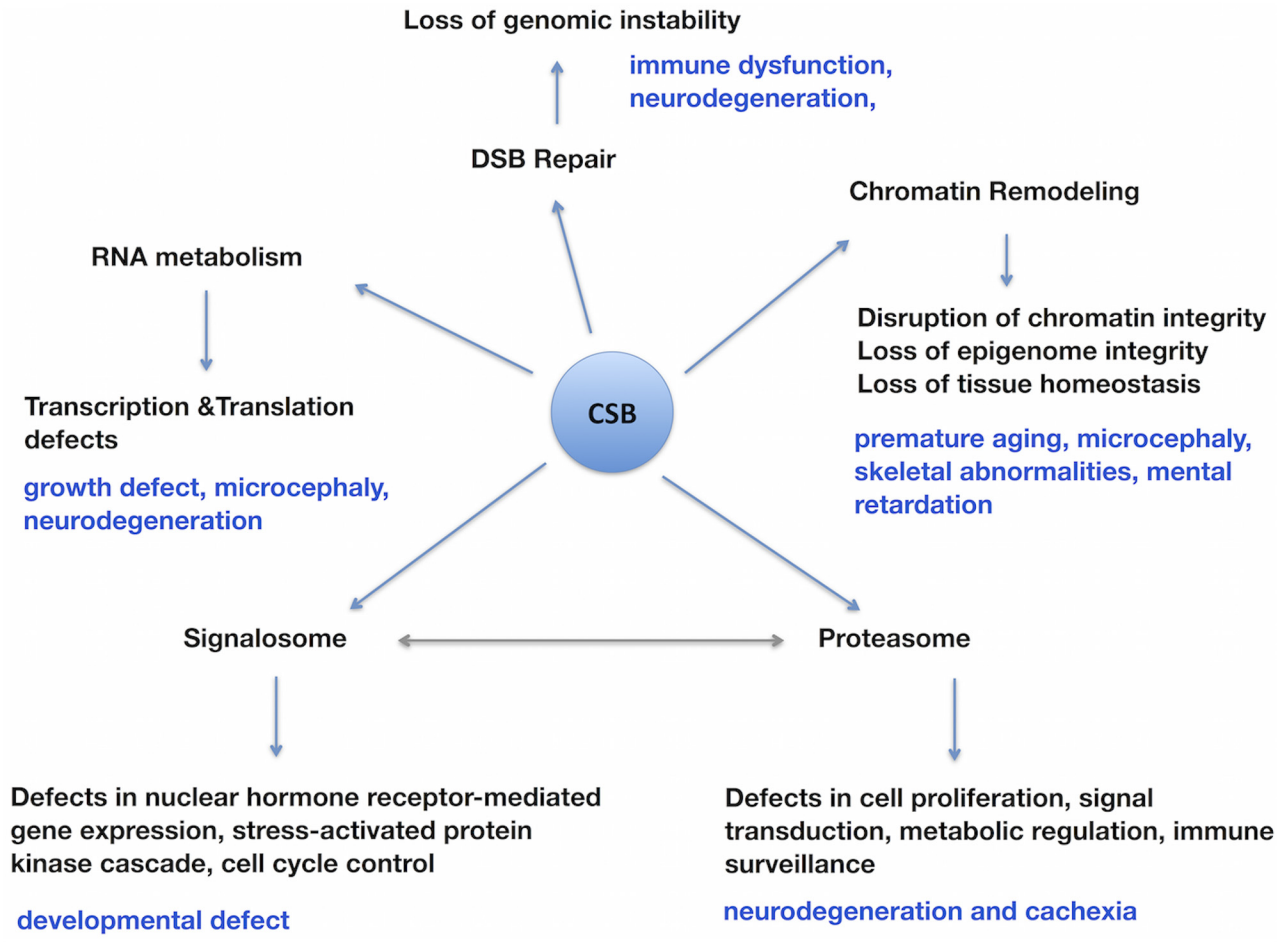
Schematic diagram illustrating the impact of functional loss of CSB on a multitude of biological processes with special relevance to some of the pathological symptoms observed in the CSB patients. Some of the pathological symptoms presumably arising due to deficiencies in various biological processes (RNA metabolism, Chromatin remodeling, DSB repair, proteasome and signalosome mediated cellular activities) due to CSB loss are indicated (blue). The double-headed arrow indicates the known interactions between signalosomes and proteasomes.

Collectively, our study has revealed a number of novel CSB co-purified proteins involved in RNA metabolism, chromatin remodeling, DSB repair, proteasome and signalosome (Fig 7). Although earlier studies have established a role for CSB in RNA polymerase elongation, our study implies a far greater role for CSB in RNA splicing and chromatin remodeling processes. Both of these are fundamental for the maintenance of tissues and organs homeostasis. Therefore, loss of CSB function in these vital processes is expected to result in degeneration of multiple organs including brain in the affected patients. Our future efforts will be directed towards validating some of the predicted functions of CSB and in developing novel therapeutic strategies to minimize the severity of some of the pathological symptoms of CSB patients.

## Materials and Methods

### Cell line

The CS1AN cell line is an SV40 transformed human fibroblast cell line, belonging to CS complementation group B lacking CSB protein [58]. This cell line represents a validated model for understanding the cellular features of Cockayne syndrome *in vitro*.

#### Construction of tagged vector, transfection and selection

The mammalian CSB expression plasmid used in the TAP technique (pZome-1-N-TAP-CSB) has been generated by inserting the full-length coding region of human CSB cDNA into the BamHI site of pZome-1-N (Euroscarf, Germany). In this construct, the TAP tag consists of the protein A (Prot.A) and the calmodulin-binding peptide affinity sequences that are separated by the recognition sequence for tobacco etch virus (TEV) protease, permitting proteolytic elution of the fusion protein from the IgG affinity resin. CSIAN (CSB deficient), cell lines was grown in DMEM/F10 medium containing 10% serum and antibiotics and the cells were transfected with either pZome-1-N (mock), or pZome-1-N- TAP-CSB using JetPEI (Polyplus) DNA transfection reagent. CSIAN cells stable expressing TAP-CSB or TAP alone were selected with puromycin (0,3 μg/ml) for 3 weeks.

#### UV survival assay

Cells were trypsinized, and 300 cells were seeded per 10-cm^2^ dish and were grown overnight. For UV treatment, cells were washed once with PBS and then irradiated at the indicated doses of UV light (254 nm). The cells were grown for 7 days, washed once in PBS, and fixed with methanol for 10 min. The fixed cells were then stained with methylene blue and washed once in PBS, and blue colonies were counted to determine the clonogenic survival of cells.

#### Western Blot analysis

Cells were lysed for 10 min on ice in RIPA buffer. The cell lysates were centrifuged at 13000 rpm for 5 min and the supernatant containing the proteins was recovered. Protein concentration was determined by Bradford protein assay kit (BioRad). Fifty micrograms of proteins were separated on polyacrylamide gradient gel (4–20%) electrophoresis and blotted onto PVDF membrane (Amersham) following a standard protocol. The membrane was incubated with TBST (20 mM Tris–HCl, pH 7.4, 137 mM NaCl; 0.2% Tween 20) buffer containing 5% NFDM for 60 min at RT and subsequently incubated with primary antibodies and HRP conjugated secondary antibody (Vector). The signal was detected using the enhanced chemiluminescence method (ECL) following the manufacturer’s instructions (Amersham).

#### Preparation of cellular extract

The cells were scraped from plates into ice-cold PBS and pelleted by centrifugation at 2000 x g for 10 min at 4°C. After the removal of excess PBS, the cell pellet (30 ml) was resuspended in 60 ml of ice-cold IPP150 lysis buffer (50 mM Tris pH 8.0, 150 mM NaCl, 10% glycerol, 0.1% NP-40, complete protease inhibitors, 1 mM PMSF). The cells were homogenized with 40 strokes in a Dounce homogenizer with a tight-fitting pestle and incubated on ice for 5 min. Insoluble material was removed by centrifugation at 16,000 x g for 20 min at 4°C.

#### Tandem affinity purification

The cell extracts were incubated with 500 μl of IgG sepharose beads for 2 h at 4°C on a rotating wheel. The IgG beads were washed twice with 60 ml of ice-cold IPP150 lysis buffer and 30 ml of TEV cleavage buffer (10 mM Tris pH 8.0, 150 mM NaCl, 10% glycerol, 0.1% NP-40, 0.5 mM EDTA, 1 mM DTT). The washed IgG beads were resuspended in 2 ml of ice-cold TEV cleavage buffer supplemented with 40 μl of AcTEV protease (400 U) and complete protease inhibitors and incubated at 16°C for 2 h on a rotating wheel. The TEV eluate was adjusted with CaCl_2_ to 3 mM final concentration, mixed with 6 ml of calmodulin binding buffer 1 (10 mM β-mercaptoethanol, 10 mM Tris pH 8.0, 150 mM NaCl, 10% glycerol, 0.1% NP-40, 1 mM imidazole, 1 mM Mg-Acetate, 2 mM CaCl_2_) and 150 μl calmodulin beads and incubated for 2 h at 4°C on a rotating wheel. The calmodulin beads were washed with 30 ml of ice-cold calmodulin binding buffer 1 and with 20 ml of calmodulin binding buffer 2 (1 mM β-mercaptoethanol, 10 mM Tris pH 8.0, 150 mM NaCl, 1 mM Mg-Acetate, 2 mM CaCl_2_). The bound proteins were eluted from beads by boiling in the LDS sample buffer, separated on a 4–12% Bis-Tris gel and visualized by silver staining.

#### Mass Spectrometry Analysis

Silver stained gel bands were used for nano-electrospray LC-MS/MS analysis. The gel bands were cut into smaller pieces and washed several times with high quality water. Disulfide bridges were reduced by dithiothreitol and alkylated by iodoacetamide. All protein samples were digested overnight at 37°C using trypsin (recombinant, proteomics grade, Roche). The digestion was stopped by acidifying to 1% with formic acid. The HPLC used was an UltiMate system (Thermo Fisher Scientific, Dionex) equipped with a PepMap C18 purification column (300μm x 5mm) and a 75μm x 150mm analytical column of the same material. 0.1% TFA was used on the Switchos module for the binding of the peptides and a linear gradient of acetonitrile and 0.1% formic acid in water was used for the elution. The LC was coupled to an LTQ (Thermo Fisher Scientific) linear ion trap mass spectrometer via the nanospray source of Proxeon (Thermo Fisher Scientific) using distal coated silica capillaries of New Objective (Woburn, MA, USA). The electrospray voltage was set to 1500V. Peptide spectra were recorded over the mass range of m/z 450–1600, MS/MS spectra were recorded in information dependent data acquisition, the default charge state was set to 2 and the mass range for MS/MS measurements was calculated according to the masses of the parent ions. One full spectrum was recorded followed by 4 MS/MS spectra for the most intense ions, automatic gain control was applied and the collision energy was set to the arbitrary value of 35. Helium was used as collision gas. Fragmented ions were set onto an exclusion list for 20 seconds.Raw spectra were interpreted by Mascot 2.2.04 (Matrix Science Ltd, London, UK) using Mascot Daemon 2.2.0. Peptide tolerance was set to +/- 2 Da and MS/MS tolerance was set to +/- 0.8 Da. Enzyme specificity was trypsin allowing 2 missed cleavages. Carbamidomethyl on cysteine was set as static modification and oxidation of methionine residues was set as variable modification. The database used for Mascot search was the nr protein database of NIH (NCBI Resources, NIH, Bethesda, MD, USA) and taxonomy was *Homo sapiens*.

#### Construction of tagged vector, transfection and co-immunoprecipitation studies

CSB-Flag tagged vector were prepared by cloning the respective ORF into the p3xFLAG-CMV-10 mammalian expression vector (Sigma-Aldrich). Protein-Myc tagged vectors were constructed by cloning the respective ORF (for each protein) into pCMV6-AC-MYC (Origene). Transient transfection was performed using X-tremeGENE DNA transfection reagent (Roche) according to the manufacturers instructions.

For co-immunoprecipitation, cell lysates (10 min on ice in RIPA buffer) from co-transfected cells were incubated with either anti-Flag M2-agarose affinity gel (A2220 Sigma-Aldrich) or anti-c-myc agarose conjugated (A7470 Sigma-Aldrich) overnight. After washing, the precipitated proteins were eluted by adding 100 μl 2× SDS-PAGE sample buffer and heating at 95°C for 10 min. The total lysates, the flow through fraction and immunoprecipitation eluates were resolved on 8% reducing SDS- PAGE gels. In some experiments, proteins were separated on gradient gels (4–20%). Blots were incubated with antibodies against Flag (F3165) and Myc (C3956) from Sigma Aldrich.

## Supporting Information

S1 Table List of proteins identified by mass spectrometry co-purifying with TAP or CSB-TAP. (XLSX)
